# Proceedings of the Second Annual Conference of the MidSouth Computational Biology and Bioinformatics Society

**DOI:** 10.1186/1471-2105-6-S2-S1

**Published:** 2005-07-15

**Authors:** Jonathan D Wren, William Slikker

**Affiliations:** 1 Advanced Center for Genome Technology, Stephenson Research and Technology Center, Department of Botany and Microbiology, 101 David L. Boren Blvd., The University of Oklahoma, Norman Oklahoma 73019, USA; 2 Deputy Director for Research, National Center for Toxicological Research, U.S. Food and Drug Administration, 3900 NCTR Road, Jefferson, Arkansas 72079, USA

**Keywords:** bioinformatics, conferences, MCBIOS, ISCB

## Abstract

The MCBIOS 2004 conference brought together regional researchers and students in biology, computer science and bioinformatics on October 7th-9th 2004 to present their latest work. This editorial describes the conference itself and introduces the twelve peer-reviewed manuscripts accepted for publication in the Proceedings of the MCBIOS 2004 Conference. These manuscripts included new methods for analysis of high-throughput gene expression experiments, EST clustering, analysis of mass spectrometry data and genomic analysis

## Introduction

The MidSouth Computational Biology and Bioinformatics Society (MCBIOS) was formed to advance the understanding of bioinformatics and computational biology by bringing together scientists of various backgrounds and disciplines and facilitating the collaboration of researchers with similar and complementary backgrounds to solve biological, health, and/or medical problems. MCBIOS also aims to promote education in bioinformatics and computational biology, informing the general public of the results and implications of current research in bioinformatics and computational biology, and promote other activities that will contribute to the development of bioinformatics and computational biology within the mid-south region of the United States [1]. MCBIOS especially supports, encourages, and mentors its student members.

The Second Annual MCBIOS conference was held at the Peabody Hotel in Little Rock, Arkansas, on October 7–9, 2004. With a unifying theme of "Bioinformatics: A Systems Approach," the conference featured three days of scientific platform presentations, posters, and panel discussions in addition to a business meeting and an Arkansas BRIN Research Symposium. Dr. Michael Gribskov, President of the International Society for Computational Biology (ISCB), and Dr. Alan Leshner, CEO of the American Association for the Advancement of Science (AAAS) and the Executive Publisher of *Science*, provided the keynote addresses. Dr. Gribskov's invigorating presentation focused on the history, development and future of systems biology and bioinformatics; Dr. Leshner's outstanding lecture on the role of science in society had the participants enthralled.

## Proceedings summary

Student platform and poster competitions were conducted and judges determined several outstanding presentations from among many excellent ones. Awards for platform (oral) presentations were given to Philip Williams of the University of Arkansas at Little Rock, Jennifer Roller of Hendrix College, and Yong Tang of the University of Arkansas at Little Rock. Awards for outstanding poster presentations were given to Yong Tang of the University of Arkansas at Little Rock, Sudeepa Bhattacharyya of the University of Arkansas at Little Rock and Phillip Romero of the University of New Orleans. These awards were underwritten by the Arkansas Biomedical Research Infrastructure Network (BRIN) program and funded through the National Institutes of Health's NCRR Division of Research Infrastructure. The US Food and Drug Administration's National Center for Toxicological Research provided additional valuable financial support.

Papers submitted for inclusion in these proceedings were peer-reviewed by two or more program committee members and external experts as necessary. The accepted papers reflect the innovative bioinformatics approaches being undertaken in the region. Several categories of research focus are apparent in the papers:

## Transcriptional Analysis using Microarrays

Zengjun "Alex" Xu and colleagues [2] employ microarray analysis in combination with a battery of bioinformatics tools and make inroads into better understanding Parkinson's Disease (PD). PD is often studied using PC12 cells, which produce dopamine, in combination with 1-methyl-4-phenylpyridinium (MPP^+^), which depletes dopamine content and elicits cell death in PC12 cells, much as is observed in PD. To identify the important genes affected in PC12 cells by MPP+, Xu *et al *identified 106 genes with differential expression levels. The genes were tied back to their ontological categories and implicated the oxidative stress and apoptosis pathways as playing a role in the observed effects. Examining these responders in terms of their literature-based associations [3], the DNA-damage pathway is identified as the likely primary culprit. Several genes are also implicated as central in this process with only loose literature ties to PD and MPP+, suggesting fruitful avenues of future experimental pursuit.

Bob Delongchamp *et al *[4] present the statistical design and analysis of a study to estimate gene expression differences between male and female livers. Addressing variation attributable to sample processing, arrays, hybridizations, normalization, and subjects, their statistical analysis suggested that about 224 genes of the 31,110 interrogated genes were expressed differentially depending upon gender. However, these differences were small and it was not possible to specify sets of differentially expressed genes that do not have large false discovery rates. The paper offers a comprehensive and statistically rigorous approach to summarizing genome-wide interrogation of gene expression changes.

Hong Fang *et al *[5] also focused upon the human liver and used a variety of bioinformatics approaches to examine microarray expression profiles from liver neoplasms that arise in albumin-SV40 transgenic rats to elucidate genes, chromosome aberrations and pathways that might be associated with human liver cancer. Their analysis implicates human chromosomes 10, 11 and 19 as regions of potential chromosomal aberrations.

Microarray-based measurements of mRNA abundance and ratio calculations assume a linear relationship between the fluorescence intensity and the dye concentration. By scanning a microarray scanner calibration slide containing known concentrations of fluorescent dyes under various PMT gains, Leming Shi *et al*. [6] demonstrated the dramatic differences in calibration characteristics of Cy5 and Cy3, indicating the importance of scanning microarrays at fixed, optimal gain settings under which the linearity between concentration and intensity is maximized. Combined with simulation results, they provided rational explanations to the existence of ratio underestimation, intensity-dependence of ratio bias, and anti-correlation of ratios in dye-swap replicates. Although normalization methods improve reproducibility of microarray measurements, they appear less effective in improving accuracy. A method of calculating ratios based on concentrations estimated from the calibration curves was proposed for correcting ratio bias.

In another paper, Leming Shi *et al *re-evaluate a study by Tan *et al *[7], which was extensively cited in a recent *Science *paper [8], that paints a very negative picture of the cross-platform comparability, and, hence, the reliability of microarray technology. Shi *et al *[9] reanalyzed Tan's dataset and found that the low cross-platform concordance reported in Tan's study appears to be mainly due to a combination of low intra-platform consistency and a poor choice of data analysis procedures, instead of inherent technical differences among different platforms. They emphasize the importance of establishing calibrated RNA samples and reference datasets to objectively assess the performance of different microarray platforms. They also discuss how the proficiency of individual laboratories can affect results as well as the merits of various data analysis procedures.

## EST Clustering

The accuracy of clustering ESTs from a large dataset representing a single species can be assessed by first clustering known genes (mRNAs) for that species to produce a non-overlapping gene set. Frank and Ercal [10] report a new algorithm for the analysis of *Glycine max *and suggest that gene family identification may be facilitated by using a hierarchical clustering method that incrementally increases the stringency of sequence matching, while Ptitsyn and Hide [11] report the design of an EST clustering program that is more efficient than its predecessors and make their software freely available to the public on an open-source basis.

In cluster analysis, however, there is no null hypothesis to test and no 'right answer'. Methodologists have suggested that the validity of clustering methods should be based on classifications that yield reproducible findings beyond chance levels. Nikhil Garge et al [12] evaluated performance of four commonly used non-hierarchical clustering algorithms (SOM, K-means, CLARA, and Fuzzy C-means) on 37 microarray datasets and found a low stability for all four algorithms even at the elevated sample sizes of n = 50. K-means showed more replicable performance than the other clustering algorithms.

## Proteomics and Mass Spectrometry

The combination of chemical crosslinking and mass spectrometry (MS) provides a powerful approach to analyze protein-protein and protein-oligonucleotide interaction sites, but the computational challenge lies in being able to effectively choose among the many different possible interpretations of MS data. Yong Tang *et al *[13] report the design of an algorithm (CLPM, for Cross-Linked Peptide Mapping) that matches peptide masses determined experimentally with theoretical peptides which could have been produced as by-products of these crosslinking reactions.

Huixiao Hong *et al *[14] report a method to reliably measure the similarities among SELDI mass spectra for quality control to decrease noise in proteomic profiling data prior to analysis. The study investigated the reproducibility of SELDI experiments and systematic variability between plates, chips, and spots on which the samples were assayed using SELDI based proteomic procedures.

## Genomic Analysis

The ever-expanding amount of genomic data provides a challenge in making observations about correlated features. Jonathan Wren *et al *[15] present an approach to standardize genomic data in a sequence matrix format and iteratively search for correlated features using Monte Carlo simulations to rearrange the features and report items with distributions that differ significantly from random allocation. Their approach is strongly limited by computational processing power and memory considerations, but the successful identification of known correlations with this approach lays the foundation for automating future exploration of genomic features.

Qian Xie *et al *[16] developed a novel adaptation of the Decision Forest pattern recognition method named Decision Forest for SNPs (DF-SNPs). Their DF-SNPs method was used to differentiate esophageal squamous cell carcinoma cases from controls, based on individual SNPs, SNP types and SNP patterns. Their method holds promise in identifying potential biomarkers from SNP data and complementing existing methods for genotype analyses.

## Summary of subchapter meetings

Since its inception, MCBIOS has tried to foster local chapters to provide more frequent contact for the membership. Currently three chapters are active: The Central Arkansas Chapter (which hosted the first two MCBIOS annual conferences), the Louisiana Chapter (which will host the next two MCBIOS annual conferences), and the Oklahoma Chapter. As one of the most active local chapters, the Oklahoma Chapter, which goes by the name of the Oklahoma Bioinformatics Society, hosted its own successful symposium, OKBIOS 2004, on November 12^th^, 2004. A total of 228 people pre-registered for the conference, 31 more registered on site, and a total of 146 people attended. Participants came from all the major Oklahoma universities, companies, government agencies, regional colleges and even a few from high schools. A very interesting group of invited speakers (9 speakers, including one who spoke through an interactive Internet videoconference) presented on a diverse range of topics, encompassing academic, government and commercial efforts in bioinformatics. More than half the participants turned in a conference evaluation form and judging from the very positive feedback, OKBIOS 2004 was a success. On a one to five scale with five being the highest satisfaction level, the average overall rating for the symposium was 4.4 (51% of participants turned in evaluations). Feedback suggested that participants especially enjoyed hearing about bioinformatics efforts and challenges from within non-academic sectors in addition to the academic talks. Vendors included ISC Bioexpress, who provided two door prizes for filling out evaluations and Silicon Genetics, who sponsored the student poster awards. In total, research groups from various Oklahoma institutions submitted 16 posters and 5 oral presentations, with 3 of those oral presentations being selected for presentation at the conference by the program committee. The program committee for OKBIOS 2004 consisted of Jonathan Wren (OU), Yuriy Gusev (OUHSC), Michelle Wiginton (OU), Sanjay Bidichandani (OUHSC), Patricia Ayoubi (OSU) and Ulrich Melcher (OSU). Organizers were invited to hold a related symposium on Synthetic Biology the day prior, which had approximately 70 participants and was also well received, making it a two-day event of bioinformatics-related symposia.

## Future Meetings

The third annual MCBIOS Conference will be held in Baton Rouge, Louisiana, on Thursday and Friday, November 17-8, 2005 at the beautiful Pennington Biomedical Research Center. The fourth annual MCBIOS Conference is scheduled to be held in New Orleans, Louisiana in 2006. Further information about MCBIOS can be found at our web site: . MCBIOS is a regional affiliate of the International Society for Computational Biology .
